# Pigmented Villonodular Synovitis of Flexor Hallusis Longus, Flexor Digitorum Longus, Tibialis Posterior: A Rare Case Report

**DOI:** 10.7759/cureus.24595

**Published:** 2022-04-29

**Authors:** Rajesh Rana, Sudarsan Behera, Chekuri Jeetendra

**Affiliations:** 1 Orthopaedics, Institute of Medical Sciences and SUM Hospital, Bhubaneswar, IND

**Keywords:** foot & ankle, pvns, tibialis posterior, flexor digitorum longus, flexor hallusis longus, pigmented villonodular synovitis

## Abstract

Pigmented villonodular synovitis (PVNS) is a locally aggressive benign tumour of the synovial membrane and tendon sheath. The unique presentation of this tumour is hemosiderin deposition with synovial proliferation. Depending on the situation, surgical excision is always the first line of treatment with adjuvant radiotherapy. Arthroscopic excision is preferred in some intraarticular nodular PVNS cases. This is a rare pigmented villonodular synovitis involving flexor hallusis longus, flexor digitorum longus, and tibialis posterior (TP). The tumour was entirely extra-articular without any joint involvement. The tumour was the diffuse type of PVNS and was treated by excision followed by radiotherapy. There is no recurrence till nine months of follow-up.

## Introduction

Pigmented villonodular synovitis (PVNS) is a rare benign condition arising from the synovial membrane of joints and tendons [1]. The first case was reported by Chassaignac in 1841 [2]. The incidence of PVNS is 1.8 per million people [3]. PVNS arises most commonly from the knee joint, followed by the hip and ankle joints. Extra-articular locations like tendon sheaths, hands, and bursae are rare [4]. Two types of PVNS are diffuse type and localized type PVNS. Localized PVNS has been well-localized like a nodule, with a clear margin for resection. Localized ones have a better prognosis with a lower recurrence rate [5]. Diffuse PVNS commonly arises from the synovium and spreads along the tendon sheath. It has a higher recurrence rate [6]. PVNS is a low-grade malignancy with various clinical presentations like a painless nodule, stiff joints, diffuse swelling around joints, and painful joints. The primary treatment protocol for PVNS is excision of the tumour, either by open synovectomy or arthroscopic excision [7]. The mode of treatment depends on the extent of growth and whether it is localized or diffuse. Localized to the joint only, cases of PVNS can be treated with arthroscopic excision with the same effectiveness as open excision [8]. PVNS arising from the tendon sheath is quite rare. Pigmented villonodular synovitis arising from the posterior ankle tendons is rare. A few cases have only been reported till now.

This is a rare pigmented villonodular synovitis arising from the synovial sheath of the flexor hallucis longus (FHL), flexor digitorum longus (FDL), and tibialis posterior (TP) tendons.

## Case presentation

**Figure 1:**
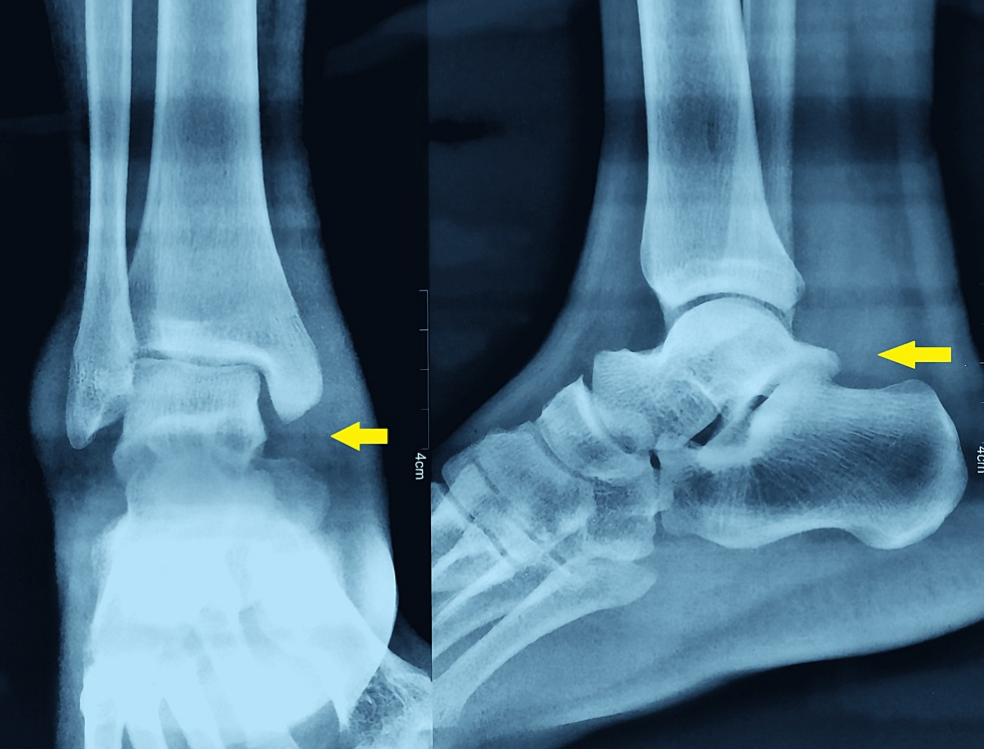
Plane radiograph of ankle and foot Ankle and subtalar joint are normal

A 17-year-old male patient presented to the hospital with a chief complaint of swelling around the right ankle for one year. The swelling was gradually increasing on the medial aspect of the ankle. The patient had a flexible flat foot bilaterally. The right foot had more exaggeration than the other foot because of the tumour affecting medial structures. The swelling was diffuse nodular, roughly around 7 cm × 14 cm extending from the navicular bone to the medial malleolus on the posteromedial aspect of the ankle. The overlying skin was normal, and there was no sinus, venous engorgement, or inflammation. On clinical examination, the ankle, joint, and subtalar joint were not involved. No other joints were found to be involved in the body. A plain radiograph of the ankle and subtalar joint showed no abnormality (Figure 1).

**Figure 2:**
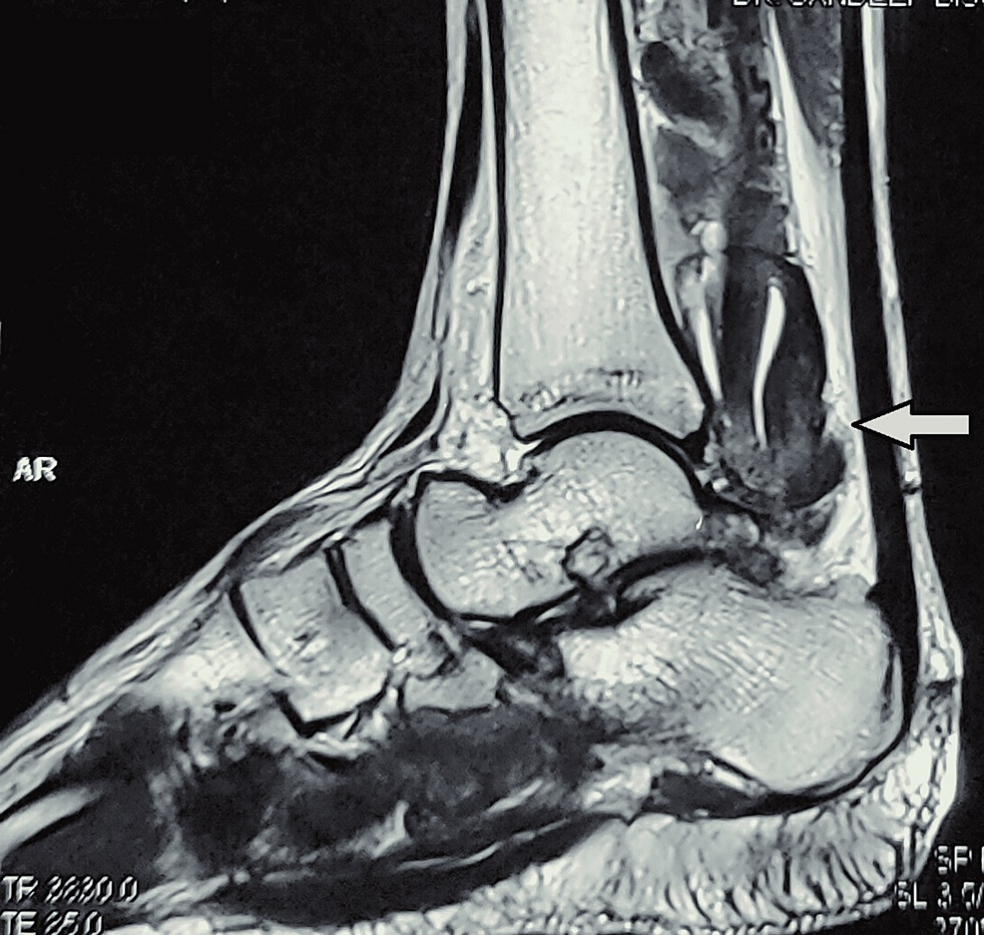
MRI of ankle and foot

MRI showed tenosynovitis with marked lobulated synovial thickening around the FHL tendon, FDL tendon, and TP tendon. Blooming was seen in gradient recalled echo (GRE) sequences of MRI, suggesting paramagnetic depositions, probably hemosiderin (Figure 2).

**Figure 3:**
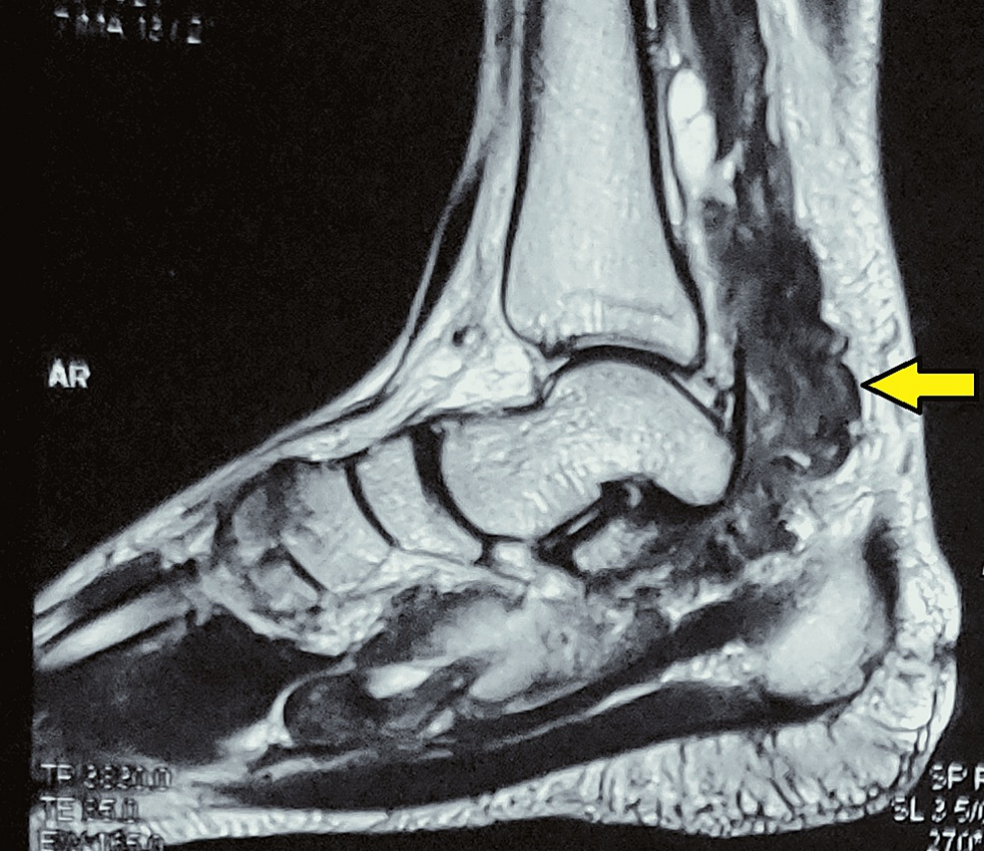
MRI sagittal section of ankle and foot Tumour extending up to midfoot

Some of the T2 weighted images were heterogeneous. Synovial thickening had extended up to midfoot. The tendons were normal in thickness and signal intensity. There was no involvement of the ankle joint. The lobulated thickening MRI features were consistent with pigmented villonodular synovitis (Figure 3).

**Figure 4:**
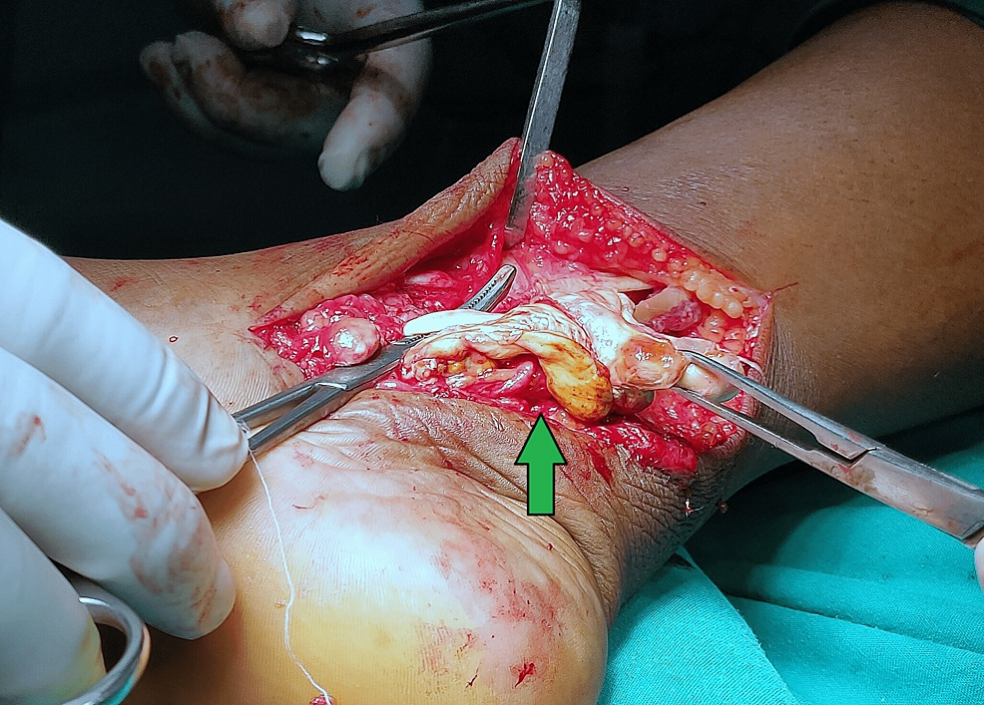
Intra-operative picture of tumour Pigmented villonodular tissues surrounding the tendons with yellowish colouration due to hemosiderin deposition

After proper evaluation and preoperative workout, we planned for the excision of the lesion. The lesions were approached through a posteromedial approach to the ankle and the three tendons. The tumour tissues were surrounding the tendons of FHL, FDL, and TP. There was no involvement of the neurovascular bundle. All the tumour tissues were excised from the midfoot area to 5 cm above the medial malleolus. The diseased tissue was nodular and brownish, surrounding the tendons (Figure 4).

**Figure 5:**
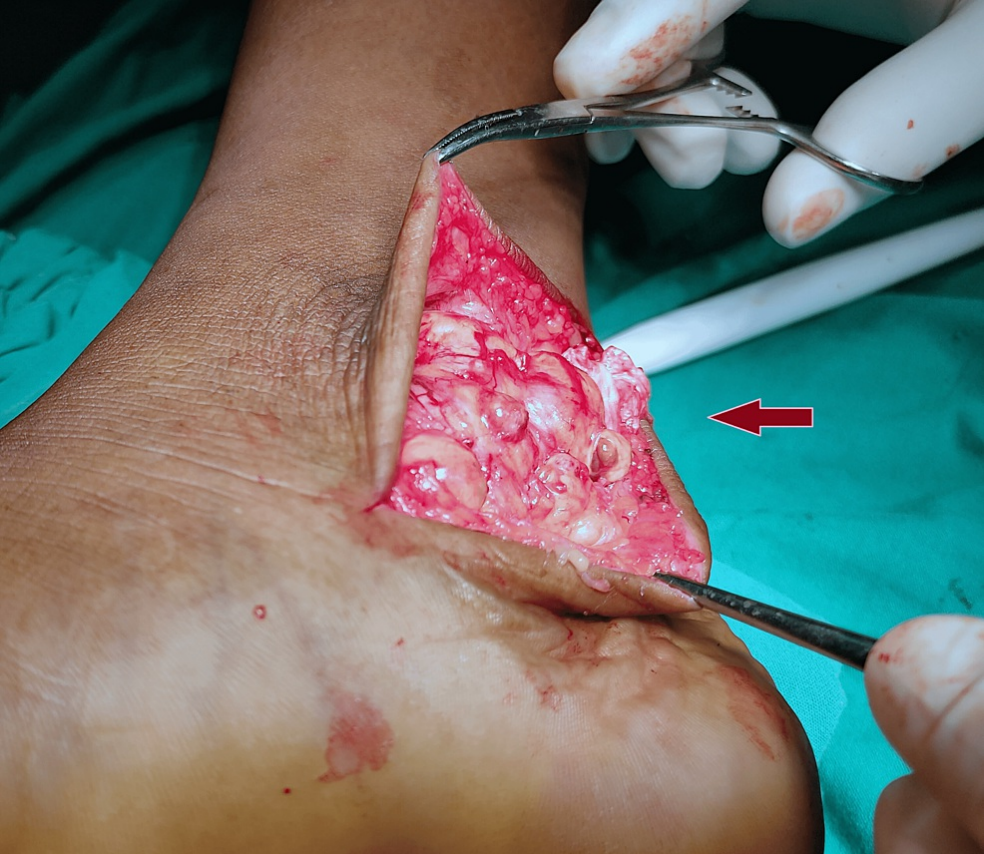
Tumour on posteromedial aspect of ankle

The tendons were found to be normal without damage from tumour tissues. The tendons were stretched due to tumour tissues but were not severe. The excised synovial tissue was sent for a histopathology examination (Figure 5).

**Figure 6:**
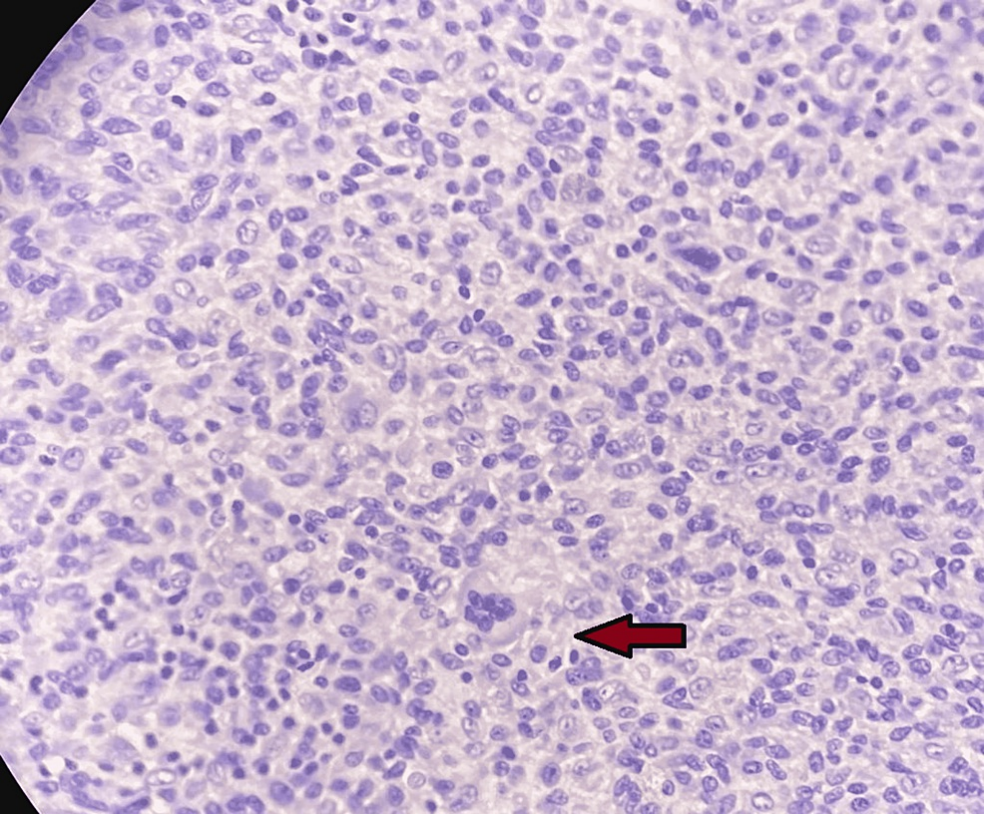
Histopathological section showing scattered giant cells

**Figure 7:**
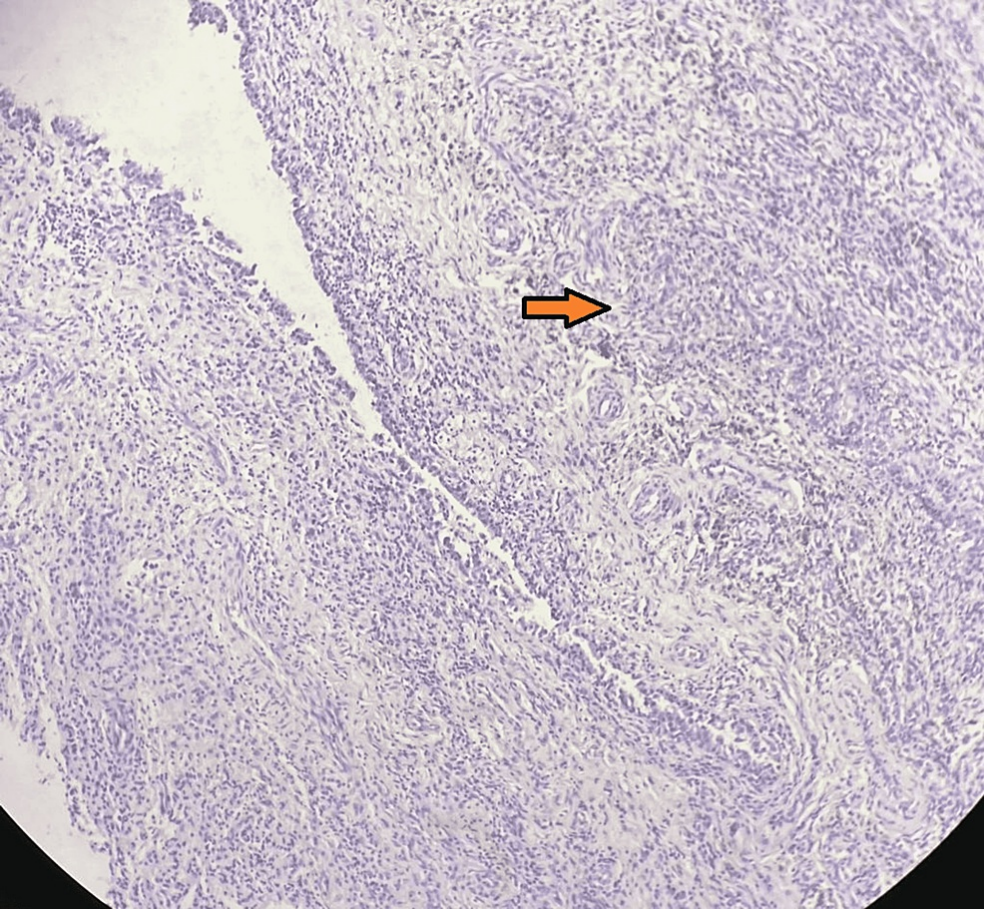
Synovial lining with underlying stroma having haemosiderin laden macrophages, granulation tissue, and mononuclear cell infiltration

Histopathological examination showed villous structures with focal hyperplasia. A synovial lining with underlying stroma had haemosiderin-laden macrophages, granulation tissue, and mononuclear cell infiltration. The stroma showed scattered giant cells. All the features were consistent with pigmented villonodular synovitis (Figures 6-7).

Post-operatively, the patient was given an ankle brace with medial arch support for the foot. Passive stretching exercises were started. Weight-bearing started after three days. Full range of motion of the ankle and subtalar joints was achieved soon without any instability. Postoperative radiotherapy was given because of the diffuse verity of the pigmented villonodular synovitis. The patient was followed up for six months without any recurrence.

## Discussion

Pigmented villonodular synovitis of the foot and ankle region grows into different joint spaces and extraarticular regions diffusely because there is no compact superficial muscle layer like in other joints [9]. Complete excision is difficult sometimes because of diffuse spread to different joint spaces. Most of the time, pigmented villonodular synovitis arises from joints and spreads extraarticular in the foot and ankle. It was completely extraarticular, surrounding TP, FHL, and FDL, which is a rare type of presentation. The diffuse type of PVNS of the foot and ankle needs post-op radiotherapy because of the difficulty of en bloc excision [10]. A moderate dose of radiotherapy is usually sufficient. Radiotherapy in benign tumours always has potential risks, like malignant transformation and wound healing problems. So radiotherapy is reserved for diffuse-type PVNS, only seeing the risk-benefit.

Guo et al. showed that pigmented villonodular synovitis of the ankle with extraarticular extension has a higher recurrence rate [5,11]. They recommended radiotherapy for these diffuse-type PVNS after surgery. Patients should be informed of the prognosis and recurrence chances before treatment in cases of diffuse-type PVNS. In their paper, Kim et al. published that the recurrence rate in diffuse PVNS is 12.5% and in nodular PVNS is around 0% [5,8]. There is always the limitation of studies with fewer cases because of the rarity of the disease.

Arthroscopic excision is an effective treatment modality because of its less morbidity and smaller incision. If the PVNS is diffuse and spread along the tendon sheath, open excision is better with less recurrence [12]. In our case, the lesion was diffuse and extraarticular along the tendon sheath. That is why we opted for open excision of the lesion. According to Roman-Ramos et al. [13], hindfoot PVNS has a better prognosis with open excision.

In feet and ankles, fusion of the affected joints reduces the chance of recurrence [14]. It is mainly preferred for osteoarthritic and eroded joints. In hindfoot PVNS cases, there is an increased risk of ankle and subtalar arthritis because of erosions and altered biomechanics due to mass effect [5]. A non-union may sometimes occur in these cases following arthrodesis. Our case was extraarticular, so we did not consider any arthrodesis procedures. Recently, imatinib and other tyrosine kinase inhibitors are being used as neoadjuvant chemotherapy [15]. Only a few studies have been done till now on it, so it is still under investigation. Imatinib is reserved for recurrent cases and PVNS of difficult locations where complete resection is difficult.

## Conclusions

Pigmented villonodular synovitis is a rare tumour arising from the joints and tendon sheath. Excision is still the primary modality of treatment. Postoperative radiotherapy is considered a diffused verity of PVNS. The risk of recurrence is more for the diffuse verity of PVNS. Chemotherapies like imatinib and tyrosine kinase inhibitors are still under investigation.
